# Spin in Randomized Controlled Trials in Obstetrics and Gynecology: A Systematic Review

**DOI:** 10.1089/whr.2021.0141

**Published:** 2022-09-20

**Authors:** Ryan Chow, Eileen Huang, Sarah Fu, Eileen Kim, Sophie Li, Jasmine Sodhi, Togas Tulandi, Kelly D. Cobey, Vanessa Bacal, Innie Chen

**Affiliations:** ^1^ Faculty of Medicine, University of Ottawa, Ottawa, Canada.; ^2^ Faculty of Biology, University of Ottawa, Ottawa, Canada.; ^3^ Department of Obstetrics & Gynecology, McGill University Health Centre, Montreal, Canada.; ^4^ Centre for Journalology, Clinical Epidemiology Program, Ottawa Hospital Research Institute, Ottawa, Canada.; ^5^ School of Epidemiology and Public Health, Faculty of Medicine, University of Ottawa, Ottawa, Canada.; ^6^ Ottawa Hospital Research Institute, The Ottawa Hospital, Ottawa, Canada.; ^7^ Department of Obstetrics and Gynecology, University of Ottawa, Ottawa, Canada.; ^8^ Clinical Epidemiology Program, Ottawa Hospital Research Institute, Ottawa, Canada.

**Keywords:** interpretive bias, obstetrics, gynecology, RCTs, methodology, reporting

## Abstract

**Objectives:**

The objective of this study was to evaluate the extent, type, and severity of spin in randomized controlled trials (RCTs) in obstetrics and gynecology.

**Data Sources:**

The top five highest impact journals in obstetrics and gynecology were systematically searched for RCTs with non-significant primary outcomes published between January 1, 2019, and December 31, 2020.

**Methods:**

Study selection and data extraction assessment were conducted independently and in duplicate. The extent, type, and severity of spin was identified and reported with previously established methodology, and risk of bias was assessed with the Cochrane Risk-of-Bias 2 Tool independently and in duplicate. Fisher's exact tests were used to evaluate the association between study characteristics, risk of bias, and spin.

**Results:**

We identified 1475 publications, of which 59 met our inclusion criteria. Articles evaluated interventions in obstetrics (*n* = 37, 63%) and gynecology (*n* = 22, 37%). Spin was not detected in 28 (47%) of the articles: Three (5%) had one, 10 (17%) had two, and 18 (31%) had greater than two occurrences of spin. Compared with articles where no spin was detected, spin was associated with the Cochrane Risk-of-Bias domain pertaining to missing data (*p* < 0.05). No association was observed with the journal, funding source, number of authors, types of interventions, and whether the study involved gynecology or obstetrics.

**Conclusions:**

Spin was detected in nearly half of 1:1 parallel two-arm RCTs in obstetrics and gynecology, highlighting the need for caution in the interpretation of RCT findings, particularly when the primary outcome is nonsignificant.

## Introduction

Results from randomized controlled trials (RCTs) constitute the highest certainty of evidence and are used to guide clinical practice.^1^ However, despite the rigorous methodology of RCTs, the language used in the reporting may not accurately reflect study findings. Unfortunately, poor-quality reporting can adversely impact subsequent research or the clinical care of patients, highlighting the importance of the appropriate reporting of results in science.^2^

Publication bias is a well-recognized phenomenon that may lead study authors to subconsciously or consciously overemphasize study findings when seeking publication. “Spin” in the context of study reporting refers to any reporting practices that result in the findings appearing more favorable than justified.^3^ Spin is a well-recognized concept in fields such as politics, where it involves the manipulation of language to influence public opinion.^4^ Within the scientific medical literature, spin has been previously assessed in RCTs in several disciplines, including cardiology, psychology, psychiatry, and, to a limited extent, obstetrics and gynecology.^2,5,6^

In cardiology RCTs, spin was found in 62 (67%) of main texts in a sample size of 93 RCTs. In psychology and psychiatry, spin was detected in 65 (56%) of the 116 included studies. Both these studies were conducted with high methodological rigor, with spin assessment conducted in duplicate. The authors concluded that spin was highly prevalent in their respective fields. However, there is very limited evidence on the occurrence of spin within the obstetrics and gynecology literature.

Given that RCTs are often considered to provide a higher level of evidence than other study designs, and that many systematic reviews and meta-analysis projects synthesize evidence from RCTs to inform the development of clinical practice guidelines, it is particularly important to evaluate, understand, and address spin in RCTs. Techniques to classify and identify the type and severity of spin in RCTs have been developed.^3^ Generally published techniques to evaluate spin examine cross-sections of published work that reports a nonsignificant primary outcome (*p* > 0.05). Boutron devised a technique to identify the extent, type, and severity of spin in RCTs with a nonsignificant primary outcome.^3^ The RCTs with a statistically nonsignificant primary are believed to be more likely subject to potential bias in reporting.

Although spin involves bias in the representation of study findings, it is distinct from bias from methodology, which is what is generally assessed when determining study quality. However, both bias in methodology and reporting may reflect a lack of attention to scientific rigor. We hypothesize that studies with more risk of bias in methodology may also be associated with more spin in the interpretation. The purpose of our study was to conduct a contemporary assessment of spin using a systematic search strategy with two independent reviewers at each stage of the study, including study selection, data extraction, and spin assessment.^6^

The primary outcome of this research was to describe the prevalence of spin in a cross-section of articles published in the field of obstetrics and gynecology. The secondary outcome was to classify where in the article spin occurs, the type of spin, and to identify the overall severity of spin among our included studies.

## Methods

The protocol was prepared in accordance with the Preferred Reporting Items for Systematic Reviews and Meta-analyses (PRISMA) protocol (PRISMA-P) and made available on Open Science Framework (OSF).^6,7^ A protocol for this study was registered *a priori* on PROSPERO, ID: CRD42020190284.

### Search strategy

The electronic bibliographic database Medline was searched on June 15, 2019 from January 1, 2019 to June 15, 2020 with the assistance of a university health sciences librarian experienced in systematic reviews for articles meeting the inclusion criteria.

We included articles published in the top five highest impact journals in the field of obstetrics and gynecology in 2019, as defined by SciMago for the year 2019.^8^ We retrieved original reports of RCTs from: *American Journal of Obstetrics and Gynecology, Obstetrics and Gynecology, Ultrasound in Obstetrics and Gynecology, Human Reproduction, and Gynecologic Oncology*.^8–13^

We included only RCTs with a 1:1 parallel two-arm study design and clearly defined primary outcomes that were statistically nonsignificant (*i.e*., *p* > 0.05). Articles were excluded if they had a predefined ineligible study design (phase 1 and 2 RCTs, factorial or split-body designs, cluster trials, equivalence or non-inferiority trials, crossover trials, multigroup trials, observational studies, case reports, or systematic reviews and meta-analyses). The strategy for RCT selection reflects those used in previous assessments of spin.^2^ Non-English reports or reports describing animal or cadaver studies were excluded.

### Study selection

Screening of titles and abstracts, as well as full texts for studies meeting eligibility criteria was performed by two independent reviewers (E.H., S.F., E.K., S.L.). Discrepancies during either stage of screening were resolved by discussion, or with a third adjudicating reviewer if needed.

Data were independently extracted, in duplicate for journal name, journal impact factor (Clarivate Analytics), year of publication, total citations (Google Scholar), funding source, and the types of study interventions.^14,15^ The Cochrane Risk-of-Bias Tool 2 was used to assess the risk of bias in included studies independently, and in duplicate.^16^

### Characteristics of included studies

Table 1 provides the characteristics of the included studies.

**Table 1.** Characteristics of Included Studies, Randomized Trials in Obstetrics and Gynecology Literature, January 2019 to December 2020

Trial characteristic	Median [range]/*N* (%)
Total	59
Journal Impact Factor	5.51 [4.62–6.12]
No. of citations up to July 2020	2 [0–13]
Year of publication	
2019^a^	30 (51)
2020^b^	29 (49)
Research area	
Obstetrics	22 (37)
Gynecology	37 (63)
Experimental arm	
Device or drug	33 (56)
Participative intervention^c^	5 (8)
Novel care pathway	21 (36)
Comparator arm	
Comparator device or drug	7 (12)
Comparator participative intervention	2 (3)
Placebo	20 (33)
Standard care	30 (51)
Funding source	
Government	9 (15)
Other public institution	28 (47)
Industry	14 (24)
Funding not stated	8 (14)
No. of authors	
1–8	40 (68)
9+	19 (32)

^a^ January 1 to December 31, 2019.

^b^ January 1 to December 31, 2020.

^c^ Initiative that focuses on patient participation, for example, exercise or education.

### Spin assessment

The extent, type, and severity of spin were identified and classified in the results and conclusions sections of abstracts, and the results, discussion, and conclusion sections of the main text in using methods previously published.^2^ This is described as follows.

### Extent

The extent of spin refers to the frequency of spin within the article. The extent of spin for each study was classified as none; Low: spin in one section other than the conclusion section; Medium: spin in the conclusion section only, or spin in two sections of the main text, not including the abstract; and High: spin identified in all sections of the main text.

### Type

The type of spin was classified into the following categories: (1) authors having pivoted to statistically significant secondary results, which could be from secondary outcomes, subgroup analyses, within-group comparisons, or per-protocol analyses; (2) authors representing nonsignificant results of primary outcomes as evidence for treatment of non-inferiority or equivalence or to rule out adverse events; and (3) authors overemphasizing the beneficial effects of the treatment without, or while acknowledging the statistically nonsignificant primary outcome. In cases where non-primary outcomes and subgroup analyses yielded statistically significant results, if the authors clearly reported that the primary outcome was nonsignificant, without trying to overshadow this or emphasize treatment efficacy based on non-primary outcomes or subgroup analyses, then the reporting of significant non-primary or subgroup results was not classified as an instance of SPIN.

### Severity

The severity, or level, of spin was classified as none, low, moderate, or high. Low spin was defined as the acknowledgment of the nonsignificant primary outcome and that future research would be beneficial. Moderate spin was defined as a lack of acknowledgment of the nonsignificant primary outcome, with some ambivalence regarding recommendations for future research. High spin was defined as a lack of acknowledgment of the nonsignificant primary outcome and no recommendations for future research.

### Statistical analysis

Characteristics of included studies and assessment of spin were expressed as categorical variables. Fisher's exact tests were used to evaluate the association between study characteristics, risk of bias, and spin.

### Protocol amendments

Following the registration of our study protocol, we did not identify any randomized trials from our search of one of our initially identified journals, *Human Reproduction Update*. As such, a decision was made to modify our methods to include the next highest impact journal, *Obstetrics and Gynecology.*^10^

## Results

### Study selection

**FIG. 1.**
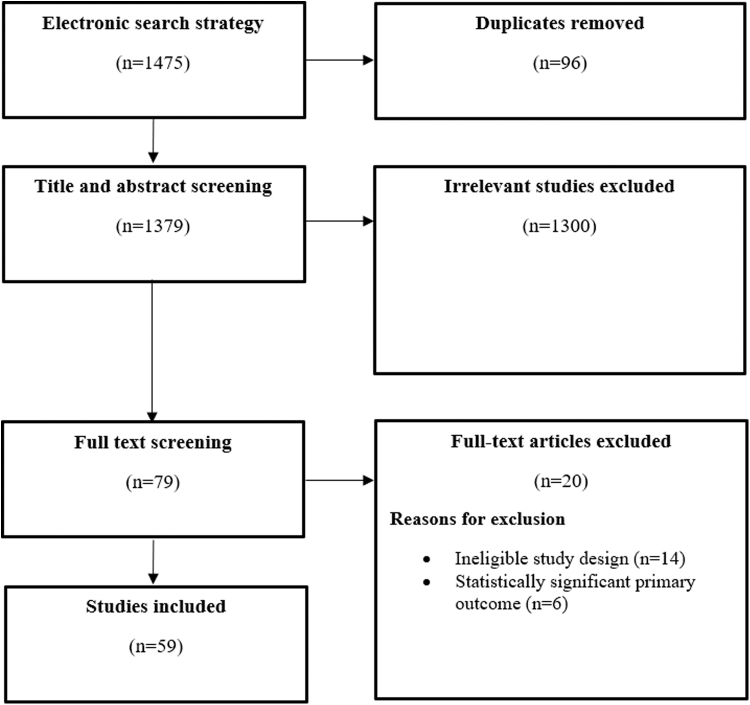
PRISMA flow diagram for study selection, randomized trials in obstetrics and gynecology literature, January 2019 to December 2020. 1475 publications were identified, of which 96 (6.5%) were duplicates. Seventy-nine (5.3%) titles and abstracts passed initial title and abstract screening, and full-text articles were retrieved. Six (0.41%) articles were excluded, as they had statistically significant primary outcomes. Fourteen (0.95%) articles were excluded, as they did not have 1:1 parallel, two-arm study designs. A total of 59 (4.0%) studies met the inclusion criteria. PRISMA, Preferred Reporting Items for Systematic Reviews and Meta-analyses.

Our search identified 1475 publications, of which 96 (6.5%) were duplicates. Seventy-nine (5.3%) titles and abstracts passed initial title and abstract screening, and full-text articles were retrieved. Search results were uploaded into the Covidence software platform (Veritas Health Innovation Ltd.). Five (0.41%) articles were excluded as they had statistically significant primary outcomes. Twelve (0.80%) articles were excluded as they did not have 1:1 parallel, two-arm study designs. A total of 59 (4.0%) articles met the *a priori* inclusion criteria and were included for analysis. See PRISMA flow in Figure 1. The list of included studies is available online on OSF.^7^

Table 2 presents the extent of spin according to various study characteristics. The prevalence of spin was not associated with the year of publication, research area, experimental or comparator arms, funding, or the number of authors. However, quality appraisal with Cochrane Risk of Bias 2 Tool (RoB2) was found to be associated with varying degrees of spin. Spin was associated with Bias due to missing data (*p* = 0.004), and non-significant trends were observed for Overall Risk of Bias (*p* = 0.06).

**Table 2.** Extent (Frequency) of Spin by Study Characteristics, Randomized Trials in Obstetrics and Gynecology Literature, January 2019 to December 2020

	Spin frequency	Spin absent	Spin present—No. of occurrence(s)		
			One	Two	Three or more
	Median, [range]	*n* (%)	*n* (%)	*n* (%)	*n* (%)
Total	0 [0–5]	28 (46)	3 (5)	10 (17)	18 (19)
Year of publication					
2019	1 [0–5]	14 (24)	2 (3)	8 (14)	6 (10)
2020	0 [0–4]	14 (22)	1 (2)	2 (3)	12 (20)
Research area					
Obstetrics	0 [0–5]	11 (19)	1 (2)	5 (8)	5 (8)
Gynecology	0 [0–4]	17 (29)	2 (3)	5 (8)	13 (22)
Experimental arm					
Device or drug	0 [0–5]	15 (25)	2 (3)	4 (7)	12 (20)
Participative intervention	0 [0–3]	3 (5)	0 (0)	2 (3)	0 (0)
Novel care pathway	1 [0–3]	10 (17)	1 (2)	4 (7)	6 (10)
Comparator arm					
Comparator device or drug	1 [0–5]	2 (3)	1 (2)	1 (2)	3 (5)
Comparator participative intervention	1.5 [0–3]	1 (2)	0 (0)	0 (0)	1 (2)
Placebo	0 [0–4]	11 (19)	0 (0)	5 (5)	4 (3)
Standard care	1 [0–3]	13 (22)	2 (3)	4 (7)	10 (12)
Funding					
Government	0 [0–3]	6 (10)	0 (0)	2 (3)	1 (2)
Other public institution	0 [0–3]	14 (24)	2 (3)	4 (7)	8 (14)
Industry	0.5 [0–4]	5 (8)	1 (2)	0 (0)	8 (14)
Funding not stated	2 [0–3]	3 (5)	0 (0)	4 (7)	1 (2)
No. of authors					
1–8	0 [0–3]	20 (34)	3 (5)	7 (12)	10 (20)
9 or more	0 [0–5]	8 (14)	0 (0)	3 (5)	8 (10)
Cochrane Risk-of-Bias Assessment					
Bias due to randomization	1 [0–4]	5 (8)	0 (0)	1 (2)	4 (7)
No bias due to randomization	2.5 [0–5]	23 (39)	3 (5)	9 (15)	14 (24)
Bias due to missing data^a^	2 [0–5]	6 (10)	2 (3)	7 (12)	6 (10)
No bias due to missing data	2 [0–5]	22 (37)	1 (2)	3 (6)	12 (20)
Bias in selection of reported outcomes	2 [0–5]	11 (19)	1 (2)	6 (10)	8 (14)
No bias in selection of reported outcomes	2.5 [0–5]	17 (29)	2 (3)	4 (5)	10 (17)
Overall risk of bias	1.5 [0–5]	18 (31)	2 (3)	9 (15)	11 (19)
No risk of bias	1 [0–2]	10 (17)	1 (2)	1 (2)	7 (12)

^a^ *p* < 0.05.

### Extent and type of spin

The extent, or frequency, of each type of spin varied between different sections of the article and is displayed in Table 3. In the results section, 13 (22%) articles were identified to have spin due to a focus on secondary outcomes. A handful of studies, six (10%), had spin due to a focus on subgroup analysis. In the discussion section of the main texts, 12 (20%) of included studies were identified to have spin due to secondary outcomes whereas 7 (12%) had spin due to a focus on subgroup analysis. The conclusion section of the included articles had the greatest frequency of spin.

**Table 3.** Type of Spin According to Section of Article, Randomized Trials in Obstetrics and Gynecology Literature, January 2019 to December 2020

Type of spin in each section	Full article, *n* (%)	Abstract, *n* (%)	Main text, *n* (%)
Results			
No spin	40 (67)	40 (67)	42 (71)
Focus on secondary outcomes	13 (22)	12 (20)	8 (14)
Focus on subgroup analysis	6 (10)	3 (5)	6 (10)
Discussion			
No spin	—	—	38 (64)
Focus on secondary outcomes	—	—	12 (20)
Focus on subgroup analysis	—	—	7 (12)
Conclusion			
No spin	32 (54)	38 (64)	3 (5)
Focus on secondary outcomes	13 (22)	8 (14)	9 (15)
Claiming treatment equivalence	6 (10)	5 (8)	3 (5)
Acknowledging nonsignificant results for primary outcome, but focusing on beneficial effect of treatment	6 (10)	3 (5)	5 (8)
Claiming efficacy despite nonsignificant results	2 (3)	1 (2)	1 (2)
Extent of spin^a^			
None	—	37 (63)	32 (54)
Low	—	11 (19)	10 (17)
Medium	—	6 (10)	5 (8)
High	—	5 (8)	11 (19)
Severity of spin^b^			
None	—	36 (61)	31 (53)
Low	—	19 (32)	20 (34)
Medium	—	1 (2)	8 (14)
High	—	3 (5)	0 (0)

^a^ Extent of spin refers to the number of sections where spin occurs. Low: spin in one section other than the conclusion section. Medium: spin in the conclusion section only, or in two sections of the main text. High: spin identified in all sections of the main text.

^b^ Severity of spin describes the severity of spin present. Low: the acknowledgment of the nonsignificant primary outcome and that future research would be beneficial. Moderate: lack of acknowledgment of the nonsignificant primary outcome, with some ambivalence regarding recommendations for future research. High: lack of acknowledgment of the nonsignificant primary outcome and no recommendations for future research.

A focus on secondary outcomes was found in nine (15%) of conclusion sections. Five (8%) studies acknowledged the nonsignificant results but focused on the beneficial effect of treatment anyway. Three (5%) of the included studies concluded that the intervention they studied was noninferior to control, despite that not being the *a priori* objective of the study. One (2%) study claimed treatment efficacy without consideration of their nonsignificant primary outcome.

### Overall severity of spin

Table 3 also outlines the severity, or level, of spin determined in each section of the included studies. Thirty-seven (63%) abstracts were determined to have no spin. Eleven (219%) abstracts had low spin, 6 (10%) had medium spin, and 5 (18%) had high spin. Thirty-two (54) main texts were determined to have no spin. The other main texts were distributed as 14 (27%) low spin, 8 (16%) medium spin, or 0 (0%) high spin.

## Discussion

In our study, we detected the presence of spin in 33% of abstracts and 40% of main texts among RCTs with nonsignificant primary outcomes published in the top five highest impact journals in obstetrics and gynecology. Although there are few comparable reports in the field of obstetrics and gynecology, these figures appear to be slightly less than those in cardiology literature, where spin was found in 62 (67%) of main texts in a sample size of 93 RCTs.^2^ In the field of psychology and psychiatry, spin was detected in 65 (56%) of the 116 included studies.^4^

Spin was detected in 53 (46%) in the conclusion sections of articles with nonsignificant primary outcomes and a 1:1 two-arm design published in 2019 and 2020. This is a noteworthy finding of concern to the obstetrics and gynecology scientific community, and it may be necessary to implement safeguards (*e.g*., researcher training, peer review protocols) in place to eliminate spin in reporting.

The most common types of spin detected (20%) involved focusing on the results for secondary outcomes and subgroup analyses in the conclusions section of the abstract or main text. The next most common type of spin (12%) detected was the emphasis or focus on a positive effect of an intervention, while still acknowledging the statistically nonsignificant result. This strategy was generally adopted when there was a statistically significant change from baseline in the intervention group of the RCT, but not compared with the control.

In our study, we also hypothesized that studies with risk of bias in methodology may also be subject to more bias in reporting. We found that the presence of spin was associated with a risk of bias in study methodology as assessed by RoB2 along the domains of overall risk of bias and bias due to missing data. Although we did not come across another study reporting the association between spin and risk of methodologic bias in the scientific literature, the findings may lend support to the notion that both risk of bias in methodology and bias in interpretation may stem from lack of attention to scientific rigor. However, more studies are needed to substantiate these findings.

The presence of spin within RCTs in high impact journals—and possible association with increased risk of methodologic bias—has important implications for future research, clinical and health policy decision making. Research indicates that clinicians reading an abstract with spin will rate the experimental treatment as more beneficial and will be more interested in reading the full text, despite assessing the trial as less methodologically rigorous.^17^

In our study, we also explored the association between the presence of spin and several other study characteristics. In agreement with other reports in the literature, we did not find an association with research area type (obstetrics or gynecology), journal, number of authors, or funding source. In addition, we did not observe an association between the presence of spin and funding source. This is consistent with the previous studies of spin and raises concerns for clinicians in the interpretation of results.^2,5^ As high rates of underreporting of financial conflicts of interest by investigators have been documented in the literature, the interpretation of these articles should be conducted with this in mind.^2^

Several strategies have been proposed to counter the effects of spin and promote higher quality reporting in science. First, awareness should be raised among researchers, peer-reviewers, clinicians, and health care decision makers on the presence of spin in the scientific literature. Although traditional critical appraisal of the scientific literature has tended to focus on detecting bias in methodology, increased attention should be paid to the detection of bias in interpretation of results. Second, spin in the medical literature may decrease if authors face less pressure to report positive findings. Publication bias is a well-recognized phenomenon, and increased recognition of the importance of negative findings may help advance science by decreasing the perceived need for spin for publication of the results.

Finally, as there are currently a very limited number of reports of spin in the obstetrics and gynecology literature, ongoing studies of this nature are necessary to audit and provide the community with feedback on temporal changes in spin. Further, we need additional studies addressing barriers and facilitators for reducing spin and to identify factors that may help identify strategies to further improve scientific reporting in this specialty.

Compared with other reports of spin in other specialties, the sample size of our study was modest. However, we believe this to be reflective of the smaller number of RCTs in obstetrics and gynecology compared with other specialties. In addition, although our study is representative of high impact journals within obstetrics and gynecology, the inclusion of only studies from the five highest impact journals reporting RCTs within our specialty limits the generalizability of our findings to other journal categories.

Further, the study only included 1:1 parallel two-arm RCTs with nonsignificant primary outcomes. Although the inclusion of this subset of randomized studies is consistent with previously published methodology on the study of spin (Boutron^3^), it is, nonetheless, a limitation on the generalizability of our study findings to other types of studies. Finally, despite our efforts to avoid bias by adhering to strict study selection and spin assessment criteria by involving two independent reviewers at all stages, the potential for residual subjective bias may still exist.

## Conclusion

Our findings suggest that spin is present in a substantial number of parallel two-arm randomized trials that have nonsignificant primary outcomes in the obstetrics and gynecology literature. As spin can influence research, clinical, and health care system decision making, the presence of such interpretive bias is of concern to the scientific community. This highlights the need to promote strategies to counter the effects of spin and promote higher quality reporting in science within the specialty.

## Authors' Confirmation Statement

All authors have approved of the article at the time of submission.

## Abbreviations Used

OSF: Open Science Framework
PRISMA: Preferred Reporting Items for Systematic Reviews and Meta-analyses
RCT: randomized controlled trial
